# Effectiveness of Telephysiotherapy in Improving Older Adults’ Physical and Psychological Outcomes: A Systematic Review and Meta-Analysis

**DOI:** 10.3390/healthcare12171775

**Published:** 2024-09-05

**Authors:** Siu-Shing Man, Huiying Wen, Kung-Ting Chiu, Fenghong Wang, Hoi-Shou Chan

**Affiliations:** 1 School of Design, South China University of Technology, Guangzhou 511436, China; ssman6@scut.edu.cn (S.-S.M.); 202320155839@mail.scut.edu.cn (H.W.); 2 Department of Systems Engineering, City University of Hong Kong, Hong Kong, China; kungtchiu2-c@my.cityu.edu.hk (K.-T.C.); alan.chan@cityu.edu.hk (H.-S.C.)

**Keywords:** telephysiothreapy, telerehabilitation, older adults, meta-analysis

## Abstract

(1) Objective: to investigate the effectiveness of telephysiotherapy compared with traditional rehabilitation for elderly patients and determine the factors impacting its efficacy. (2) Method: Five online databases (PubMed, Google Scholar, Scopus, Web of Science, and Cochrane) were reviewed up to 31 July 2023. The search included the literature in English from 2014 to 2023, to capture the latest practices in telephysiotherapy for elderly patients. Data from all qualified studies were independently extracted by two authors, quantifying effect size to reflect treatment performance. (3) Results: 222 records from 19 articles were analyzed. The effect size for telephysiotherapy was 0.350 (95% C.I. = 0.283–0.416; *p* < 0.01). The standardized mean differences for physical and psychological outcomes were 0.406 (95% C.I. = 0.325–0.488; *p* < 0.01) and 0.223 (95% C.I. = 0.110–0.336; *p* < 0.01), respectively. (4) Conclusions: Telephysiotherapy was more effective than traditional rehabilitation, significantly improving the physical and psychological status of elderly patients. The factors influencing the effectiveness of telephysiotherapy were intervention type, intervention duration, outcome, and gender.

## 1. Introduction

The unprecedented growth of the aging population has drawn increased attention to the older population [1]. In China, nearly 20% of the population is over the age of 60, and they face significant challenges in terms of cognitive, motor, sensory, and mental health. More than 78% of older people have one or more chronic diseases [2]. In the UK, the proportion of the population aged 65+ has risen from 16.4% in 2011 to 18.6% in 2021 [3]. Similarly, in the United States, where 16% of the population were 65 or older in 2019, there has been a significant increase in the number of people with chronic diseases such as dementia, heart disease, and diabetes [4]. This global aging trend presents significant health challenges that require proactive measures and appropriate care to mitigate adverse effects.

Physical therapy and rehabilitation are essential to improve physical and self-care abilities, disease progression, and functional capacity in older patients [5]. Traditional physiotherapy includes therapeutic exercises, activities, and treatments such as joint manipulation and mobilization [6]. It is essential for pain relief, improved function, and quality of life in patients with musculoskeletal disorders [7] and enhances mobility in patients with subacute stroke [8]. However, the COVID-19 pandemic has severely disrupted face-to-face physiotherapy services [9,10]. Time and financial constraints, coupled with regional disparities in rehabilitation services, particularly affect older people with limited mobility or in remote areas [11]. As a result, there is a growing need for alternative treatment modalities to address these limitations and meet the rehabilitation needs of patients.

Telephysiotherapy utilizes information and communication technology (ICT), such as videoconferencing or telephone services [12], to allow patients to be treated at home [13,14]. It includes rehabilitation, assessment, monitoring, prevention, and counseling [15]. Rehabilitation is particularly important for increasing daily mobility and improving quality of life, especially for older adults with mobility impairments [16,17]. Ahmadi Marzaleh et al. [11] found that the use of telephysiotherapy increased significantly during the COVID-19 period. Telephysiotherapy can improve the physical performance of older people and increase their satisfaction, especially those suffering from COVID-19. Telephysiotherapy is a promising solution to overcome the limitations of traditional physical therapy and provide effective treatment services. Telephysiotherapy has been shown to be effective for a variety of conditions such as heart failure [18,19,20], stroke [21,22], falls [23,24,25], osteoarthritis [26,27,28], hip fracture [29,30], musculoskeletal problems [31,32,33], and cognitive impairment [34,35,36]. Studies have shown that telephysiotherapy is highly effective in improving fall efficacy, activities of daily living, balance, dyspnea management, physical activity, and quality of life in patients with a variety of health conditions [18,23,29]. Advances in technologies such as virtual reality and augmented reality have further expanded the scope of telephysiotherapy [37,38].

Despite the large body of experimental evidence suggesting that telephysiotherapy is beneficial for older patients, systematic reviews and meta-analyses are needed to fully understand its effectiveness and influencing factors. Most reviews have focused on specific medical conditions. For example, Nacarato et al. [39] found that cardiovascular telephysiotherapy improved cardiorespiratory health and quality of life in older adults. Cacciante et al. [40] reported that cognitive telephysiotherapy was as effective as traditional face-to-face methods for treating neurological disorders. Jirasakulsuk et al. [41] noted that older patients with musculoskeletal diseases showed significant improvement in physical functioning.

However, these reviews did not quantitatively synthesize the efficacy of telephysiotherapy for older patients through meta-analysis. In addition, the factors affecting its efficacy remain undetermined. This study aims to address these shortcomings by assessing whether telephysiotherapy is more effective than traditional rehabilitation for elderly patients and identifying the factors that influence its efficacy. The results of the study will advance the practice of telephysiotherapy in geriatric care and provide a theoretical basis for future improvements.

## 2. Materials and Methods

### 2.1. Literature Search and Screening

We identified relevant literature suitable for this study after searching and screening five online databases (PubMed, Google Scholar, Scopus, Web of Science, and Cochrane). The literature covered spanned the period from 2014 to 2023 (as of 31 December), aiming to provide a comprehensive overview of the latest practices and developments in telephysiotherapy for older patients, while ensuring that sufficient literature has been collected over the past decade. In summary, the keywords used to search and filter the literature included the following: ‘effectiveness’ OR ‘effect’ AND ‘telerehabilitation’ OR ‘telemedicine’ OR ‘telehealth’ OR ‘web-rehabilitation’ AND ‘telephysiotherapy’ OR ‘telephysical therapy’ AND ‘elderly’ OR ‘older’ OR ‘older person’ OR ‘older people’. Multiple inclusion criteria for further screening are shown below:

- The literature had to examine the efficacy of telephysiotherapy in the rehabilitation of elderly patients. Therefore, literature unrelated to telephysiotherapy was excluded. This study included studies in which patients were older than 65 y [42];
- The content of the literature had to include at least one report of the practice and monitoring of telephysiotherapy with elderly patients, including the duration of training and rehabilitation interventions, and be related to physical therapy. Articles with incomplete data were excluded;
- Studies included at least one method of comparing telephysiotherapy with physical therapy and demonstrated the effectiveness of telephysiotherapy for older adults. Studies that derived training effects only by comparing before and after tele-rehabilitation were excluded [43];
- Test data such as sample size, influential variables, and *p*-values were required to substantiate the effectiveness of treatment with telephysiotherapy. Therefore, we eliminated articles with incomplete data.

**Figure 1:**
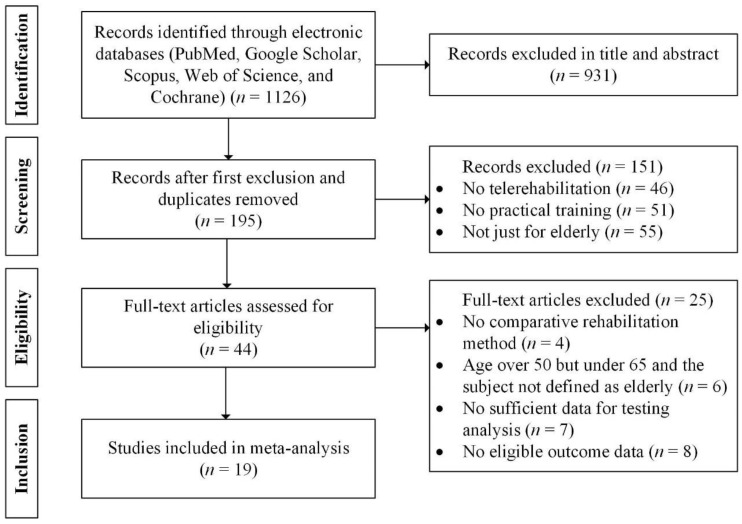
PRISMA flowchart of the study screening process.

From electronic databases, 1126 articles were initially retrieved. Nineteen articles that met the inclusion requirements were selected after screening. This review followed the Preferred Reporting Items for Systematic Reviews and Meta-Analyses (PRISMA) statement [44] (Supplementary Materials). The specific selection process can be seen in Figure 1.

### 2.2. Data Extraction and Coding

Studies that satisfied all the inclusive criteria are summarized in this report. Two authors independently completed data extraction for all eligible studies. This study quantified the effect size as a measure of the impact of telephysiotherapy on treatment outcomes in elderly patients. This study used effect size to analyze the intervention effects based on the calculations of the mean score, standard deviation, and sample size for the intervention group (IG) and control group (CG) [45]. For data analysis, the author information, publication year, sample size, demographic characteristics (such as mean age and gender), intervention type, intervention duration, and outcome were extracted from each study. Effect sizes were separately documented and calculated for different subgroups, including gender and intervention duration, within these studies. A resolution was sought from a third investigator regarding any disagreements regarding data extraction. Table 1 illustrates the coding details.

To analyze the effects of demographic characteristics and study methodology on telephysiotherapy effectiveness for older patients, moderator variables such as intervention type, intervention duration, outcome, and gender needed to be coded so that this information could be systematically categorized. Intervention types were categorized into six types according to experimental descriptions: operational management systems, software, real-time platform, telerehabilitation exercise, virtual reality telerehabilitation, and mHealth programs. According to the ratio of the intervention durations in the included studies (intervention durations of four weeks and twelve weeks accounted for 36.84% and 47.37% of all intervention durations), intervention duration was classified into three groups: ≤4, 4–12, and ≥12. The outcomes were divided into two categories: physical and psychological. Physical outcomes included musculoskeletal system, balance, cardiorespiratory, quality of life, pain and sensory. The psychological outcomes included cognition and emotion. Detailed categorization can be found in Table 2. Gender was grouped based on three ratios: only female (M) (M/F = 0), more males than females (0 < F/M < 1), and more females than males (F/M > 1). Specific codes are presented in Table 3.

### 2.3. Risk of Bias in Individual Studies

Upon reviewing the methodological information and study outcomes, two authors assessed the methodological quality of the included studies using the Physiotherapy Evidence Database (PEDro) scale [63]. The PEDro scale consisted of 11 items: eligibility criteria, random allocation, concealed assignment, baseline comparability, blinding of participants, blinding of therapists, blinding of evaluators, outcome measures assessed in 85% of participants, intention-to-treat analyses, between-group comparisons, point estimates, and variability. Excluding the initial eligibility criterion, the maximum PEDro scale score was 10. A total score of 3 or less indicated poor methodological quality [63]. Any discrepancies in the assessment were resolved by involving a third investigator.

### 2.4. Meta-Analysis

Comprehensive Meta-Analysis 3.0 was employed to analyze all statistical data, following the steps. Due to the differences in demographics, treatment methods, outcomes, and results between studies, random-effect models were used to observe the distribution of effect sizes. Furthermore, heterogeneity measures (*Q* and *I^2^*) were used to calculate statistical differences and measure heterogeneity among studies. If there was heterogeneity between studies, meta-regression analysis was performed to identify potential moderators of heterogeneity. To assess the reliability of the findings and to detect publication bias, the Begg test and Egger test were applied [64,65]. According to the conclusion by Pelletier et al. [66], the test power of Egger’s test is slightly higher than that of Begg’s test, which is more valid and responsive for small samples. Moreover, Egger’s test is often used in conjunction with fail-safe N-numbers and funnel plots to assess publication bias [67].

## 3. Results

### 3.1. Overall Effect Size

**Figure 2:**
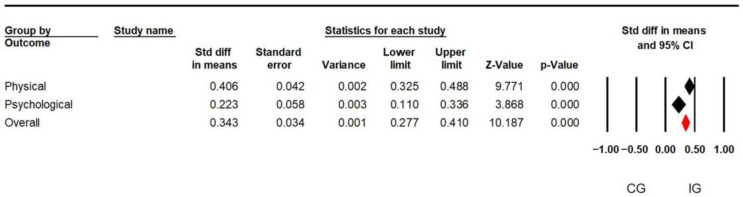
Forest plot grouped by outcomes using a mixed-effects analysis. Note: The black diamonds indicate the combined effect sizes for each outcome (physical or mental), and the red diamonds indicate the overall combined effect sizes for all outcomes. The center of the diamond is the overall SMD, and its width is the confidence interval.

In total, 222 records were retrieved from 19 articles that reported testing the efficacy of telephysiotherapy in elderly patients in physiotherapy. Table 4 shows an overall effect size of 0.350 (95% C.I. = 0.283–0.416; *p* < 0.01) in elderly patients undergoing telephysiotherapy. Further meta-analysis distinguished between physical and psychological effects, with a standard mean difference (SMD) of 0.406 (95% C.I. = 0.325–0.0.488; *p* < 0.01) for physical and 0.223 (95% C.I. = 0.110–0.336; *p* < 0.01) for psychological (see Figure 2). The findings indicate that telephysiotherapy is superior to traditional methods. Moreover, the physical and psychological outcomes were significantly improved by telephysiotherapy.

### 3.2. Test of Heterogeneity

Table 4 presents the outcomes of the heterogeneity test within the random effects meta-analysis. As a measure of heterogeneity, *Q*-value can be easily affected by the number of studies. Instead, the *I*^2^ value adjusts the *Q* value so that it does not fluctuate as the number of studies increases or decreases. When *I*^2^ exceeds 50%, it indicates the presence of moderate heterogeneity [68]. In this analysis, *I*^2^ was 61.437%, pointing to significant heterogeneity among the study data.

### 3.3. Quality of the Studies

The methodological quality of the included studies ranged from 4 to 8 on the PEDro scale, indicating a low risk of bias [63]. Table 5 shows the detailed classification.

### 3.4. Test and Adjustment for Publication Bias

Publication bias refers to the tendency for statistically significant findings to be more frequently reported and published compared w non-significant or inconclusive results [69]. This phenomenon suggests that only when small sample studies show significant large effects are these results considered meaningful, while smaller or moderate effects tend to be easily ignored. Hence, investigating publication bias and verifying the integrity of studies involves examining the correlation between sample size and effect size. To assess publication bias, we applied Begg and Mazumdar’s rank correlation test [70] and Egger’s regression intercept test [67] in this study. The results of the meta-analysis showed a statistically significant Kendall’s tau b value of 0.235 (two-tailed *p*-value less than 0.01) in the Begg and Mazumdar test, which reflected a remarkable correspondence between sample size and effect size. In addition, the Egger regression test also revealed the appearance of publication bias, with an intercept coefficient of 0.790 and a two-tailed *p*-value of less than 0.01.

One of the effective tools used to evaluate publication bias is the classical fail-safe N [71]. This approach involves determining the number of unpublished studies required to render the combined effect size of published studies statistically nonsignificant. It is computed using the formula 5n + 10, where n denotes the number of studies incorporated in the meta-analysis. If the resultant fail-safe N significantly exceeds this value, it suggests that the influence of unpublished studies on the reported results is negligible. In this study, the calculated fail-safe N was 5490, which far exceeded 1120 (222 × 5 + 10), suggesting that the findings of this study were scarcely affected by unpublished studies, indicating minimal influence from publication bias.

**Figure 3:**
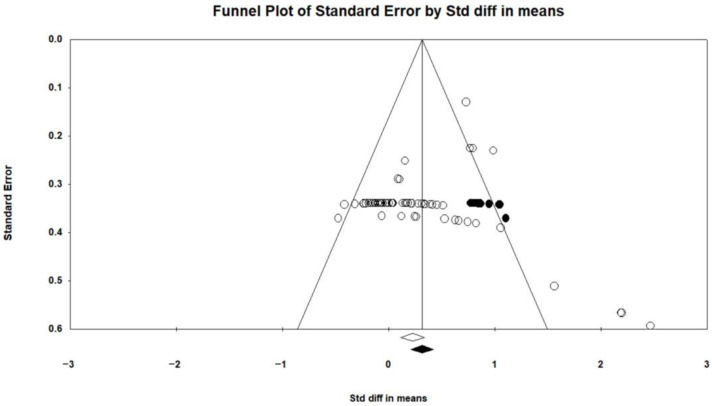
Funnel plot illustrating the psychological records post-correction for publication bias. Note: White circles represent studies or data points that show no significant bias. Black circles represent studies or data points required to show corrected unbiased or significant heterogeneity. Open circles indicate the actual observed data points, while the solid circles indicate the data points that were filled in after estimation. The open diamonds on the funnel plot show an estimate of 0.223 (95% C.I. = 0.110–0.336) for observed points, while the solid diamonds show an estimate of 0.315 (95% C.I. = 0.207–0.423) for filled points.

The trim-and-fill method can estimate unbiased effect size [72]. According to the conclusion stated by Shi and Lin [73], the overall effect size of the random effects model was kept at 0.350 (95% CI = 0.283–0.416). Additionally, out of the total 67 psychological outcome records, 11 adjustment points were included to the right of the mean following the trimming method. This adjustment led to a rise in the overall effect size based on random effects, from 0.223 (95% CI = 0.110–0.336) to 0.315 (95% CI = 0.207–0.423) (refer to Figure 3). No adjustments were necessary for the physical outcome records. After correcting publication bias, the SMD was 0.347 for rehabilitation between the IG and the CG. Telephysiotherapy was more effective than traditional rehabilitation in the two types of outcomes. Specifically, the SMDs of telephysiotherapy were 0.406 and 0.315 above traditional rehabilitation in terms of physical and psychological outcomes, respectively.

### 3.5. Moderator Analysis

The records analyzed in this study displayed moderate heterogeneity as indicated by the outcomes of the heterogeneity test reported in Section 3.2. Meta-regression analysis was used to explore potential differences in all records by analyzing a single covariate. The potential moderators were the outcome, intervention type, intervention duration, gender, and mean age. Specifically, categorical variables included outcome, intervention type, intervention duration, and gender with dummy coding; the mean age was an integral variable.

Table 6 presents the outcomes of single covariate meta-regression utilizing random effects and maximum likelihood methods for the overall records. The regression coefficient serves as a metric to gauge the extent of dependence of the dependent variable on the independent variable within regression analysis. As shown in Table 6, intervention type (*Q* = 75.75; *p* < 0.01), intervention duration (*Q* = 20.52; *p* < 0.01), outcome (*Q* = 25.58; *p* < 0.01), and gender (*Q* = 23.47; *p* < 0.01) significantly influenced the effect size, while no significant moderating effect was found for mean age (*Z* = −0.96; *p* = 0.335). The corresponding *R*^2^ values for intervention type, intervention duration, outcome, and gender were 0.46, 0.08, 0.21, and 0.16, respectively. For the intervention type, the effect size increased by 0.555 when the telerehabilitation exercise was changed to the operational management system. The effect size decreased by 0.256 and 0.394 when the software was changed to the virtual reality program. When intervention duration (weeks) changed from ≥12 to ≤4 and 4–12, the effect sizes were increased by 0.241 and 0.416, respectively. Effect sizes increased by 0.454 and 0.460 when the outcomes changed from cognitive to emotional and musculoskeletal. However, no significant difference was identified among balance, cardiorespiratory, pain, quality of life, or sensory. As for gender, the effect sizes increased by 0.161 and 0.648 when moving from 0 < F/M < 1 to F/M > 1 and M/F = 0.

## 4. Discussion

Through a meta-analysis, this study successfully verified the efficacy of telephysiotherapy compared with traditional physical therapy for elderly patients and identified the factors that affect the efficacy of telephysiotherapy for elderly patients. The results were in line with the majority of prior studies that have compared the effectiveness of traditional rehabilitation therapy with that of telephysiotherapy for elderly patients, showing that telephysiotherapy can serve the elderly patient population as a treatment method or an adjuvant therapy for traditional physical therapy. Generally, traditional rehabilitation methods are mainly based on face-to-face diagnosis and treatment services. Moreover, the physician’s recommendations are passed on by phone or orally: thus, the patients are required to see a doctor offline. Limited by the treatment scenarios and the patient’s motor function level, the therapeutic effect of patients of this traditional rehabilitation is finite. Telephysiotherapy can provide medical care services from a distance through existing communication technologies, allowing patients to perform rehabilitation training at home or other convenient places at any time. Telephysiotherapy can also ensure the continuity and effectiveness of rehabilitation training through online consultation. This study compared traditional physiotherapy and telephysiotherapy for elderly patients. Through data analysis, it is concluded that telephysiotherapy is more effective than traditional physiotherapy for older adults.

In the context of telephysiotherapy for elderly patients undergoing physiotherapy, this study offers three distinct contributions to the existing research. Firstly, it conducted a comparison of the extent and direction of outcome disparities between older patients undergoing telephysiotherapy and those receiving traditional physiotherapy. Secondly, this study found a medium effect of heterogeneity affecting the difference in effectiveness between telephysiotherapy and traditional physiotherapy in the training process. Therefore, it may be meaningless to generalize that the effects of telephysiotherapy differ from those of conventional physiotherapy. In some situations, traditional physiotherapy may be superior to telephysiotherapy. However, this claim may be misinformed in other circumstances. Thirdly, this study recognizes various factors affecting the effectiveness of telephysiotherapy. Specifically, the effectiveness of telephysiotherapy was affected by intervention type, intervention duration, outcome, and gender.

### 4.1. Effectiveness of Telephysiotherapy for Elderly Patients

Nineteen comparative studies of traditional physiotherapy and telephysiotherapy for elderly patients were analyzed. The aggregated effect sizes suggested that telephysiotherapy was more efficacious than traditional physiotherapy for elderly patients. In particular, telephysiotherapy surpassed traditional physiotherapy in physical and psychological outcomes. Specifically, the physical outcomes included physical training content such as balance, cardiorespiratory, musculoskeletal, pain, quality of life, and sensory aspects. The psychological outcomes included psychological training content, such as cognitive and emotional outcomes. For physical outcomes, Ortiz-Piña et al. [56] compared a 12-week multidisciplinary telephysiotherapy program with home-based on-site rehabilitation. Elderly patients who received telephysiotherapy significantly improved balance and musculoskeletal function in measures of functional independence and timed up-and-go tests. According to Kikuchi et al. [20], elderly patients who received 12 weeks of home-based cardiopulmonary rehabilitation with a remote real-time monitoring system had significantly improved exercise tolerance in a six-minute walk. During the COVID-19 pandemic, Bagkur et al. [74] designed an 8-week telephysiotherapy exercise program for elderly patients. According to tests, the participants experienced higher levels of physical activity and improved sleep parameters, significantly increasing their quality of life. An et al. [26] compared elderly patients who underwent a preoperative telephysiotherapy program with total knee arthroplasty and those who received usual care and underwent a 3-week intensive exercise program. The results reported by An et al. [26] showed that patients’ scores for the Western Ontario and McMaster Universities’ osteoarthritis pain index after the telephysiotherapy program were significantly higher than those following a traditional rehabilitation program. Other researchers [75] found that, after eight weeks of balance training, elderly patients who received vibration-tactile sensation enhancement at home had significantly improved sensory-organization test scores, increased vestibular dependence, and improved sensory attributes, further improving balance ability. Regarding psychological outcomes, Nousia et al. [47] conducted intervention training for 15 weeks for an IG using RehaCom telephysiotherapy and a CG receiving clinical care. The results showed that elderly patients who received telephysiotherapy intervention experienced statistically significant improvements in all domains of global cognitive performance. Menengïç et al. [54] divided patients with early Alzheimer’s disease into an IG that received real-time motor cognitive therapy and a CG that did not receive any intervention. Through the six-week post-training test, patients who received telephysiotherapy had significantly improved cognitive performance and reduced emotional symptoms such as anxiety and depression. These findings illustrated the effectiveness of telephysiotherapy for older adults.

### 4.2. Moderating Variable

#### 4.2.1. Intervention Type

Meta-regression analysis revealed significant differences in the effectiveness of telephysiotherapy between intervention types. Specifically, the operational management system had a significantly greater impact on the telephysiotherapy’s effectiveness than the telerehabilitation exercise. In contrast, software and virtual reality rehabilitation were less effective than telerehabilitation exercises. Hwang et al. [76] and Cottrell et al. [77] found that home telerehabilitation interventions (especially combined with operational management) were more effective in improving patient compliance and treatment outcomes than other approaches (e.g., software or VR programs). This finding suggests that interventions including operational management systems are particularly effective in improving patient outcomes. This comprehensive management approach helps ensure consistent and systematic treatment, resulting in increased effectiveness. Although technologies based on software and virtual reality rehabilitation interventions are innovative, their effectiveness may be affected by patients’ acceptance of the technology, ease of use, and the degree of personalization. If software and virtual reality rehabilitation interventions are not designed to be user-friendly or personalized, they may result in low patient engagement and ineffective treatment results. Interestingly, Laranjo et al. [78] found that mHealth interventions in physical therapy were able to improve patient engagement and adherence. However, the effectiveness of mHealth interventions varied depending on the specific application and design. This finding suggests that the effects of mHealth rely on the program’s design quality, patients’ technological literacy, and the intervention’s continuity. In contrast, the real-time platform intervention had no significant effect. This result may have been due to technical limitations or lack of personalization that failed to improve treatment outcomes for patients.

These findings highlight the importance of intervention type in determining the effectiveness of telephysiotherapy, with operational management systems showing higher effectiveness and software and virtual reality rehabilitation being associated with lower effectiveness than telerehabilitation exercise. The need for research to optimize and standardize these approaches should be stressed. Given the differences in results across intervention types, it is clear that a “one-size-fits-all” approach may not be appropriate for telephysiotherapy. Future research should focus on refining and testing these specific interventions to understand their effects on patients’ physical and psychological outcomes.

#### 4.2.2. Intervention Duration

This study demonstrated that the effectiveness of telephysiotherapy is optimized within an intervention period ranging from 4 to 12 weeks, surpassing outcomes achieved in interventions lasting less than 4 weeks or exceeding 12 weeks. Moreover, interventions spanning four to twelve weeks exhibited slightly superior effectiveness compared to those lasting less than four weeks. The results indicate that intervention durations less than 12 weeks is more beneficial for telephysiotherapy.

In a comparative experiment by Zhang et al. [48], hip fracture patients participating in telephysiotherapy were found to have gradually improved musculoskeletal outcomes, as evidenced by improved Harris hip scale scores and measurements of functional independence. Notably, the musculoskeletal outcomes after three months of training showed significant superiority over those after only one month. Conversely, Manenti et al. [58] conducted a comparative study involving elderly patients with mild cognitive impairment, analyzing the effectiveness of an in-person cognitive virtual reality rehabilitation system against conventional in-person cognitive therapy. Participants were segmented into three groups and evaluated at baseline, one month, four months, and seven months of the rehabilitation. The results indicated that the virtual reality rehabilitation group improved over the clinical group in the safe open forward task from baseline to one month into rehabilitation. However, their performance worsened after one month to four months. Comparing the performance of elderly cognitively impaired patients after 15 weeks of telephysiotherapy and conventional rehabilitation, Nousia et al. [47] demonstrated that, although cognitive outcomes (neuropsychological performance) improved significantly with telephysiotherapy, there were no significant differences in recall, word recognition, digit span forward/backward, or trail-making tests. The results of this study suggest that telephysiotherapy proves more effective than traditional rehabilitation across diverse treatment parameters. However, over time, its efficacy tends to align with or even diminish in comparison to traditional rehabilitation methods. These outcomes may be influenced by the specific treatment modalities examined in the literature and the duration of the experimental intervention. Future studies should encompass a wider array of rehabilitation treatments and more comprehensive data on rehabilitation interventions, through evaluation and documentation.

#### 4.2.3. Outcomes

The results indicated that the emotional and musculoskeletal rehabilitation effects were significantly higher compared to cognitive rehabilitation effects. Nevertheless, no statistically significant relationships were observed between balance, cardiorespiratory, pain, quality of life, and sensory aspects. This finding suggests that telephysiotherapy is more effective in emotional and musculoskeletal rehabilitation than in balance, cognitive, cardiorespiratory, pain, quality of life, or sensory intervention. However, a randomized controlled study by Tekin and Cetisli-Korkmaz [51] found that a 4-week home fitness program delivered via telephysiotherapy reduced depressive symptoms and fear of falling and improved standing and balance function in older adults. After the test by Kikuchi et al. [20], the cardiorespiratory function (six-minute walking distance) of older adults significantly improved (*p* = 0.003), increasing muscle strength in the lower extremities (*p* = 0.022). However, cardiorespiratory function (systolic and diastolic blood pressure) did not reach statistical significance. As a result, the combined cardiorespiratory function effect became nonsignificant. Given the combination of outcomes from various treatment types in the included studies, categorizing and analyzing the detailed outcomes for each treatment type were challenging. Future research needs to categorize and analyze the detailed outcomes of each treatment type and explore the degree of association between the outcomes in relation to the effectiveness of telephysiotherapy, to avoid ambiguous conclusions about particular outcomes.

#### 4.2.4. Gender

Within gender, the effect of telephysiotherapy was significantly higher in F/M > 1 and M/F = 0 compared with 0 < F/M < 1. Specifically, the effectiveness of telephysiotherapy for M/F = 0 was higher than that for F/M > 1. This result suggests that all-female and female-dominated telephysiotherapy is more effective than male-dominated telephysiotherapy. Poggesi et al. [79] compared the functional outcomes of males and females discharged from a rehabilitation hospital after a stroke. The results indicated that females showed better functional recovery than males. However, Rossi-Izquierdo et al. [25] designed vestibular rehabilitation with a follow-up period of 12 months to assess the factors affecting the rehabilitation outcomes of elderly patients with histories of falls. The results showed that gender did not affect vestibular rehabilitation outcomes. Despite the divergent results of previous studies on the impact of gender on telephysiotherapy’s effectiveness, the findings of this study confirm that telephysiotherapy is more effective for female patients than male patients. This result was the same as the results reported by Mao et al. [80], in that cognitive activities were more beneficial for older female patients than older male patients. A possible reason for this phenomenon is gender socialization. Males avoid seeking help, to appear strong [81]. Furthermore, it may be because only a few studies have examined the effectiveness of gender on telephysiotherapy. The conclusions about the influences of gender on traditional rehabilitation may not apply to telephysiotherapy. Further research in the future is warranted to investigate the relationship between gender and the efficacy of telephysiotherapy.

#### 4.2.5. Mean Age

The previous literature on the influence of age on telephysiotherapy’s effectiveness is contradictory. Whitney et al. [82] found that age did not affect recovery in patients after vestibular schwannoma resection when they examined vestibular rehabilitation. However, Kanyılmaz et al. [83] stated that older age in patients may lead to less improved outcomes in a short rehabilitation period. In the present study, the age of older patients did not significantly affect the overall effect size of telephysiotherapy. The lack of significance in these results could be attributed to the scarcity of the literature and the inadequate representation of age groups. As the functional status of the elderly declines with age, the way traditional physiotherapy is performed needs to change accordingly. Future studies must delve into the correlation between different age groups and telephysiotherapy effects.

### 4.3. Theoretical and Practical Implications

#### 4.3.1. Theoretical Implications

Reviewing the included research, prior studies comparing telephysiotherapy and traditional physiotherapy were found to have several methodological limitations. Firstly, when conducting randomized controlled trials, previous studies did not include much information about adverse events while patients were undergoing telephysiotherapy or the reasons for withdrawing from telephysiotherapy interventions. The lack of descriptions and summaries of the causes of incidents is detrimental to refining the functional applicability of telephysiotherapy for older patients or other populations and designing effective therapeutic tools for telephysiotherapy. Future research needs to document relevant information for improving telephysiotherapy services. Secondly, some of the literature included in this study reported small sample sizes. For example, the sample sizes of the studies by Bao et al. [61] and Giesbrecht and Miller [59] were 12 and 18, respectively. Smaller sample sizes may cause random variability and imprecision in experimental results, which may lead to heterogeneity in studies [84]. Future research must consider recruiting as many eligible people as possible when conducting relevant telephysiotherapy trials, to expand the sample size and avoid controversy in the subsequent experimental process and results. Thirdly, most studies did not explore telephysiotherapy for patients with two or more disease types. Few studies have explored the effects of telephysiotherapy on two or more associated diseases (comorbidities), for example, cardiorespiratory rehabilitation in patients with diabetes [85] or musculoskeletal rehabilitation in patients with cognitive impairment [86]. In telephysiotherapy research, the coverage for types of diseases in the elderly population is still insufficient. The potential of telephysiotherapy for older adults has also not been fully exploited. Thus, more research is required in the future to explore telephysiotherapy in groups with multiple illness types. Fourthly, according to the meta-analysis findings, intervention type, duration of intervention, outcome, and gender moderated the effectiveness of telephysiotherapy. Therefore, in the future, telephysiotherapy design for older adults should consider the type of intervention, final treatment outcome, patient’s gender, and duration of the rehabilitation intervention. Finally, the acceptance of telephysiotherapy for older adults is still not well understood. Shalabi et al. [87] found that only 19% of respondents had heard of telephysiotherapy. Krishnan et al. [88] also reviewed the challenges patients may encounter when undergoing telephysiotherapy. More research efforts should be made to examine the acceptance of telephysiotherapy in older adults.

Notably, some challenges are still encountered when implementing telephysiotherapy for older patients [89]. Firstly, remote resolution of emergencies poses certain risks when serious adverse events occur, and safety measures must be taken [90]. The second is the achievability of telephysiotherapy. Given various technical reasons, patients’ low skill levels and education levels may hinder their use of telephysiotherapy [91]. There is a need for technical setup and a trial run of the telephysiotherapy with the help of family members and therapists. Additionally, personal reasons among therapists, such as poor technical skills, efficacy, learning ability, and resistance to change in clinical practice, can be barriers to implementing telephysiotherapy [91]. Therefore, therapists must be responsive to technological changes and improve their competence. Thirdly, patient criteria for telephysiotherapy have not been sufficiently examined in the literature. Although telephysiotherapy is not inferior to traditional physiotherapy, not all patients are suitable for participation in telephysiotherapy [92], such as those with poor discipline, inability to adhere to routine exercise, and lack of confidence in the outcome of telephysiotherapy [93]. Therefore, patients need to engage in careful communication and questioning with their therapists to determine the mode of physiotherapy to be conducted. In contrast, traditional physical therapy still has advantages that cannot be replaced by telephysiotherapy at present, such as face-to-face communication and timely feedback, providing a good treatment environment and atmosphere and building patient trust and compliance. More research is also needed to suggest better improvements or implementable technology models for telephysiotherapy, to improve the existing deficiencies.

#### 4.3.2. Practical Implications

This study confirms that telephysiotherapy for elderly patients is more effective than traditional physiotherapy. Therefore, the healthcare industry is encouraged to use telephysiotherapy technology for rehabilitation training. Presently, policies around the globe are working to promote the development of telemedicine [94]. The “Internet + Healthcare” document issued by China proposed that the Internet and other information technologies should be fully utilized to provide patients with remote home rehabilitation guidance services [95]. The Hong Kong Government released has its 2023–2024 healthcare budget, in which the Chinese Medicine Development Fund provides support for discharged patients and rehabilitated persons through telemedicine consultation and rehabilitation treatment [96]. The National Institutes of Health have reported that the progress made in telemedicine during the pandemic allowed its widespread adoption, which could lower barriers to treatment and benefit pain and substance abuse treatment [97]. These policies and the current situation indicate the popularity and spread of telemedicine in health care. In the future, telephysiotherapy will also continue to be used in various medical rehabilitation scenarios.

This study demonstrated that the efficacy of telephysiotherapy for elderly patients was influenced by intervention type, intervention duration, the assessed outcomes, and gender, but not by mean age. Based on these findings, recommendations are made to promote telephysiotherapy for older patients. Firstly, government and software developers should develop operational management systems for telephysiotherapy that are suitable for physiotherapists and patients, and they should improve the ease of use of these systems [41]. Physiotherapists must develop appropriate telephysiotherapy programs for older patients based on their circumstances, with interventions ideally lasting no more than 12 weeks, after which offline-assisted rehabilitation can be performed. Secondly, physiotherapists must provide patients with real-time advice on their health status to help them improve their physical functioning and increase their confidence, to ensure positive outcomes in their treatment programs [41]. Thirdly, female elderly patients experience better rehabilitation outcomes when undergoing telephysiotherapy. Healthcare associations should pay more attention to the health status of male elderly patients and collaborate with relevant software developers to design a care telephysiotherapy system segment for male elderly patients. Finally, given that the average age of elderly patients does not significantly impact rehabilitation outcomes, the telephysiotherapy system applies to the elderly population. Based on these suggestions, clinical practice guidelines could recommend telephysiotherapy as a preferred mode of treatment for older adults, prioritizing interventions under 12 weeks and focusing on mood and musculoskeletal rehabilitation. To overcome potential barriers, healthcare providers should address issues such as access to technology and patient education and ensure the continuity of care through regular follow-ups and support. The government and social groups need to strengthen care and assistance for the elderly, promote Internet diagnosis and treatment services, implement the well-being of the elderly through policies, optimize the process of smart medical services, and improve the patient’s experience [95].

### 4.4. Limitations

Firstly, 19 articles extracted for this study was a small number. Some previous studies could not be used to extract effect sizes due to incomplete reporting of information. Consequently, the research data may have been subject to uncertainty, leading to a reduction in generalizability of the findings for industry. Future studies need to report their results in detail, and more research is required to investigate the effectiveness of telephysiotherapy for older persons. Secondly, given various experimental designs in the included studies, subjective categorization of the moderating variables (e.g., intervention type) may have led to biased findings. Future research on telephysiotherapy for elderly patients requires a detailed description of the research design to reduce possible results bias. Third, telephysiotherapy outcomes were meticulously categorized based on physical and psychological for balance, cardiorespiratory, cognitive, emotional, musculoskeletal, quality of life, pain, and sensory. However, the categorization of outcomes was divided by primary function (e.g., functional independence measures were categorized as musculoskeletal) and did not strip away the different functional characteristics of the outcomes. Subsequent investigations ought to offer a comprehensive description of the functional attributes of outcomes, to facilitate effective categorization.

## 5. Conclusions

This meta-analysis investigated 222 records from 19 articles over the past decade to assess the effectiveness of telephysiotherapy for older adults. The results showed that telephysiotherapy was more effective than traditional physical therapy, with physical and psychological treatments being 0.406 and 0.315 points more effective, respectively. Significant associations (*p* < 0.01) were found between intervention duration, rehabilitation outcome, and patient gender and the effectiveness of telephysiotherapy. Compared with telerehabilitation exercise, operational management systems had significantly higher effectiveness of telephysiotherapy, while software and virtual reality rehabilitation were less effective. Interventions lasting less than 12 weeks were more effective. Improvements in emotional and musculoskeletal rehabilitation were more significant than balance, cognition, cardiorespiratory, pain, quality of life, and sensory outcomes. In addition, female older patients had better rehabilitation outcomes than males. These findings support the wider use of telephysiotherapy in medical rehabilitation.

## Figures and Tables

**Table 1:** Summary of included studies in this systematic review and meta-analysis.

Author (Year)	Sample Size	Mean Age (± S.D.)	Gender (F/M)	Condition	Control Group	Intervention Type	Intervention Duration	Timepoint	Outcomes
Wu et al. (2023) [46]	85 (IG = 43; CG = 42)	IG: 74.28 ± 5.06; CG: 72.00 ± 6.77	IG: F/M > 1; CG: F/M > 1	Hip fracture	Usual care	Operative management system	25 weeks	Baseline; 4, 12, and 25 weeks	Musculoskeletal, emotional
Nousia et al. (2023) [47]	30 (IG = 15; CG = 15)	IG: 75.73 ± 4.48; CG: 76.67 ± 3.81	IG: 0 < F/M < 1; CG: 0 < F/M < 1	Cognitive impairment	Clinical care	Software	15 weeks	Baseline; 15 weeks	Cognitive
Zhang et al. (2022) [48]	51 (IG = 27; CG = 24)	IG: 77.00 ± 7.89; CG: 75.17 ± 7.73	IG: F/M > 1; CG: F/M > 1	Hip fracture	Usual care	Operative management system	12 weeks	2, 4, and 12 weeks	Musculoskeletal, balance
Tsai et al. (2022) [49]	81 (IG = 40; CG = 41)	IG: 75.6 ± 6.0; CG: 73.3 ± 5.0	IG: 0 < F/M < 1; CG: 0 < F/M < 1	Heart failure	Usual care	Telerehabilitation exercise	25 weeks	Baseline; 25 weeks	Cardiorespiratory
Torpil et al. (2022) [50]	48 (IG = 24; CG = 24)	IG: 68.25 ± 3.32; CG: 68.91 ± 2.56	IG: F/M > 1; CG: F/M > 1	Total knee arthroplasty	Clinical care	Real-time platform	4 weeks	Baseline; 4 weeks	Quality of life, pain, emotional, cognitive, musculoskeletal
Tekin et al. (2022) [51]	255 (IG = 132; CG = 123)	IG: 68.34 ± 4.33; CG: 70.34 ± 5.37	IG: 0 < F/M < 1; CG: 0 < F/M < 1	Fall risk	Exercise	Telerehabilitation exercise	4 weeks	4 weeks	Balance
Tao et al. (2022) [52]	71 (IG = 38; CG = 33)	IG: 66.6 ± 7.5; CG: 63.2 ± 9.1	IG: F/M > 1; CG: F/M > 1	Lower limb amputation	Usual care	Software	12 weeks	Baseline; 4, 9, and 12 weeks	Balance
Mora-Traverso et al. (2022) [53]	64 (IG = 30; CG = 34)	IG: 75.77 ± 5.67; CG: 80.38 ± 5.54	IG: F/M > 1; CG: F/M > 1	Hip fracture	Usual care	Real-time platform	12 weeks	Baseline; 12 weeks	Quality of life, pain, emotional, musculoskeletal
Menengïç et al. (2022) [54]	20 (IG = 10; CG = 10)	IG = 77.7 ± 5.29; CG = 80.6 ± 6.11	IG: F/M > 1; CG: F/M > 1	Alzheimer’s disease	Usual care	Real-time platform	6 weeks	Baseline; 6 weeks	Cognitive, balance, quality of life, musculoskeletal, emotional
Li et al. (2022) [29]	31 (IG = 15; CG = 16)	IG: 76.5 ± 8.6; CG: 82.1 ± 9.7	IG: F/M > 1; CG: F/M > 1	Hip fracture	Exercise	Software	15 weeks	12, 15 weeks	Balance, musculoskeletal, pain, quality of life
Yerlikaya et al. (2021) [55]	34 (IG = 18; CG = 16)	IG: 70.22 ± 5.53; CG: 71.81 ± 6.57	IG: F/M > 1; CG: F/M > 1	Balance	Exercise	Telerehabilitation exercise	8 weeks	8 weeks	Balance, emotional, quality of life
Ortiz-Piña et al. (2021) [56]	62 (IG = 28; CG = 34)	IG: 75.86 ± 5.79; CG: 80.38 ± 5.54	IG: F/M > 1; CG: F/M > 1	Hip fracture	Usual care	Telerehabilitation exercise	12 weeks	12 weeks	Musculoskeletal, balance
Arena et al. (2021) [57]	144 (IG = 72; CG = 72)	IG: 76.6 ± 7.0; CG: 77.2 ± 8.2	IG: F/M > 1; CG: F/M > 1	Fall risk	Exercise	Telerehabilitation exercise	12 weeks	Baseline; 12 weeks	Cardiorespiratory, balance
An et al. (2021) [26]	36 (IG = 18; CG = 18)	IG: 71.1 ± 3.30; CG: 70.38 ± 2.59	IG: M/F = 0; CG: M/F = 0	Total knee arthroplasty	Usual care	Telerehabilitation exercise	6 weeks	Baseline; 3, 6 weeks	Musculoskeletal, balance, pain, sensory
Manenti et al. (2020) [58]	25 (IG = 18; CG = 17)	IG: 75.3 ± 3.3; CG: 78.1 ± 4.1	TL: 0 < F/M < 1 (IG: 0 < F/M < 1; CG: F/M > 1)	Cognitive impairment	Clinical care	Virtual reality rehabilitation	30 weeks	Baseline, Baseline; 12, 17, and 30 weeks	Cognitive, quality of life
Giesbrecht et al. (2019) [59]	18 (IG = 10; CG = 8)	TG: 65.0 ± 8.6	IG: 0 < F/M < 1; CG: 0 < F/M < 1	Wheelchair skills training	Clinical care	mHealth program	4 weeks	4 weeks	Musculoskeletal, quality of life
Bernocchi et al. (2019) [60]	112 (IG = 56; CG = 56)	IG: 71 ± 9; CG: 70 ± 9.5	IG: 0 < F/M < 1; CG: 0 < F/M < 1	Heart failure	Clinical care	Telerehabilitation exercise	17 weeks	Baseline, 17 weeks	Cardiorespiratory, quality of life
Bao et al. (2018) [61]	12 (IG = 6; CG = 6)	IG: 76.2 ± 5.5; CG: 75.0 ± 4.7	TL: F/M > 1 (IG: F/M > 1; CG: F/M = 1)	Balance	Usual care	mHealth program	8 weeks	Baseline, 4, 8 weeks	Balance, sensory
Hong et al. (2017) [62]	23 (IG = 11; CG = 12)	IG: 82.2 ± 5.6; CG: 81.55 ± 4.4	IG: F/M > 1; CG: F/M > 1	Sarcopenia	Usual care	Telerehabilitation exercise	12 weeks	12 weeks	Musculoskeletal

Note: S.D. = standard deviation. IG: intervention group; CG: control group; TG: total group; M/F = 0: only female; 0 < F/M < 1: more males than females; F/M > 1: more females than males.

**Table 2:** Detailed classification of function tests in outcomes.

Classification	Test Details
Musculoskeletal system	Harris hip scale, functional independence measure, Nottingham health profile (energy and physical activity), fitness level (general, strength, speed and flexibility), grip strength, extension peak torque, knee flexion range of motion, Western Ontario and McMaster Universities osteoarthritis index (function), wheelchair skills test, wheel control, wheelchair outcome measure, arm curls, back scratches, chair sit-and-reach and eight-feet up-and-go.
Balance	Timed up-and-go test, short physical performance battery, modified fall efficacy scale, 2 min walk test, four-step square test, activities balance confidence, five times sit-to-stand test, one-leg stand test, functional reach test, Morse fall scale, Berg functional balance scale, sway, four-stage balance test, STEADI fall risk, HOME FAST.
Cardiorespiratory	Six-minute-walk distance, fitness level (cardiorespiratory), systolic/diastolic blood pressure, heart rate, Medical Research Council dyspnoea scale, Minnesota living with heart failure questionnaire score, chronic obstructive pulmonary disease assessment test.
Quality of life	Canadian occupational performance measure, Nottingham health profile (sleep), quality of life (self-care, daily activities), Katz activities of daily living scale, Zarit caregiver burden inventory, modified Barthel index, Lawton instrumental activities of daily living scale, World Health Organization quality-of-life instrument, life-space assessment, and health utility index
Pain	Nottingham health profile [pain], quality of life (pain), visual analogue scale, pressure pain threshold, Western Ontario and McMaster Universities osteoarthritis index (pain).
Sensory	Sensory organization test, somatosensory reliance, visual reliance, and vestibular reliance.
Cognition	Word recognition, delayed memory, digit span forward/backward, Boston naming test, semantic fluency, trail-making test, clock-drawing test, Montreal cognitive assessment, Nottingham health profile (social isolation), geriatric depression scale, mini-mental-state examination, Warwick–Edinburgh mental well-being scale, everyday memory, Rey auditory verbal learning test, immediate/delayed recall, free and cued selective reminding test, immediate/delayed recall/index of sensitivity of cueing, verbal fluency—phonemic/semantic, battery for analysis of aphasic deficits, objects/actions naming.
Emotion	Self-rating anxiety scale, Nottingham health profile (emotional reactions), quality of life (anxiety), Beck anxiety scale, and trait anxiety inventory.

**Table 3:** Information on the coding of the moderating variables.

Variable	Category	Coding Method
Outcome	Physical and psychological outcomes	Physical: musculoskeletal system, balance, cardiorespiratory, pain, quality of life, and sensory; Psychological: cognition and emotion.
Gender	M/F = 0, 0 < F/M < 1, F/M > 1	M/F = 0: Only females; 0 < F/M < 1: More males than females; F/M > 1: More females than males.
Intervention type	Operational management systems, software, real-time platform, telerehabilitation exercise, virtual reality rehabilitation, and mHealth programs	Operational management systems: hip fracture post-operative management system; Software: RehaCom software 6.10.2, Wii Fit activities, and mobile app; Real-time platform: online platform (Zoom 4.3.0, Skype 8.125.0.20, or Microsoft Teams 10.4.7) for real-time treatment; Telerehabilitation exercise: real-time monitoring of each exercise session and interactive telerehabilitation home exercise; Virtual reality rehabilitation: home-based cognitive virtual reality rehabilitation treatment system; mHealth program: delivering healthcare-related services through mobile devices.
Intervention duration	≤4, 4–12, ≥12	≤4: Intervention duration less than or equal to 4 weeks; 4–12: Intervention duration between 4 and 12 weeks; ≥12: Intervention duration more than or equal to 12 weeks.

**Table 4:** A random effects meta-analysis of the comparison between the intervention group (IG) and control group (CG).

Effect Size and 95% Confidence Interval					Null Test (Two-Tailed)		Heterogeneity			
Number of records	Point estimate	Standard error	Variance	95% C.I.	Z-value	*p*-value	*Q*-value	df(*Q*)	*p*-value	I^2^ (%)
222	0.350	0.034	0.001	(0.283–0.416)	10.307	0.000	573.095	221	0.000	61.437

**Table 5:** PEDro scale for the included studies.

Author (Year)	1*	2	3	4	5	6	7	8	9	10	11	Score
Nousia et al. (2023) [47]	1	1	0	1	0	0	0	1	1	1	1	6
Wu et al. (2023) [46]	1	0	0	1	0	0	1	1	1	1	1	6
Tao et al. (2022) [52]	1	1	0	1	0	0	0	1	1	1	1	6
Mora-Traverso et al. (2022) [53]	1	0	0	1	0	0	1	1	1	1	1	6
Li et al. (2022) [29]	1	1	0	1	0	0	1	1	1	1	1	7
Menengïç et al. (2022) [54]	1	1	1	1	0	0	0	1	1	1	1	7
Tsai et al. (2022) [49]	1	0	0	0	0	0	0	1	1	1	1	4
Torpil et al. (2022) [50]	1	1	1	1	0	0	1	1	1	1	1	8
Tekin and Cetisli-Korkmaz. (2022) [51]	1	1	0	1	0	0	0	1	1	1	1	6
Zhang et al. (2022) [48]	1	1	0	1	1	0	0	1	1	1	1	7
Yerlikaya et al. (2021) [55]	1	1	1	1	0	1	0	1	1	1	1	8
Arena et al. (2021) [57]	1	1	1	1	0	0	1	1	1	1	1	8
Ortiz-Piña et al. (2021) [56]	1	0	0	0	0	0	1	1	1	1	1	5
An et al. (2021) [26]	1	1	1	1	1	0	1	1	1	1	1	9
Manenti et al. (2020) [58]	1	1	1	1	0	0	1	1	1	1	1	8
Bernocchi et al. (2019) [60]	1	1	1	0	0	0	1	1	1	1	1	7
Giesbrecht et al. (2019) [59]	1	1	1	1	0	0	0	1	1	1	1	7
Bao et al. (2018) [61]	1	1	1	1	0	1	0	1	1	1	1	8
Hong et al. (2017) [61]	1	1	0	1	0	0	0	1	1	1	1	6

Note: 1* = eligibility criteria specified; 2 = random allocation of participants; 3 = concealed assignment; 4 = baseline comparability; 5 = participant blinded; 6 = therapist blinded; 7 = accessor blinded; 8 = outcome measures assessed in 85% of participants; 9 = intention-to-treat analysis; 10 = between-group statistic comparisons; 11 = point estimates and variability.

**Table 6:** Overall records of meta-regression with random effects and maximum likelihood methods.

Covariate	Coefficient	Z-Value	2-Sided *p*-Value	Test to Model	R^2^
Intervention type ** (refer to telerehabilitation exercise)					
Intercept	0.420	7.98	0.000	/	0.46
mHealth program	0.174	1.45	0.148	*Q* = 75.75, *df* = 5, *p* < 0.001	
Operational management system **	0.555	4.71	0.000		
Real-time platform	−0.017	−0.17	0.866		
Software **	−0.256	−3.00	0.003		
Virtual reality rehabilitation **	−0.394	−4.76	0.000		
Intervention duration (week) ** (reference to ≥12)					
Intercept	0.242	5.66	0.000	/	0.08
≤4 *	0.214	2.55	0.011	*Q* = 20.52; *df* = 2; *p* < 0.001	
4–12 **	0.416	4.21	0.000		
Outcome ** (reference to cognitive)					
Intercept	0.166	2.66	0.008	/	0.21
Balance	0.130	1.49	0.136	*Q* = 25.58; *df* = 7; *p* < 0.001	
Cardiorespiratory	0.178	1.31	0.191		
Emotional *	0.454	2.51	0.012		
Musculoskeletal **	0.460	4.65	0.000		
Pain	0.210	1.17	0.243		
Quality of life	0.165	1.33	0.184		
Sensory	0.333	1.60	0.109		
Gender ** (reference to 0 < F/M < 1)					
Intercept	0.213	3.94	0.000	/	0.16
F/M > 1 *	0.161	2.31	0.021	*Q* = 23.47; *df* = 2; *p* < 0.001	
M/F = 0 **	0.648	4.76	0.000		
Mean age					
Intercept	0.920	1.56	0.119	/	0.01
Mean age	−0.008	−0.96	0.335	Z = −0.96; *p* = 0.335	

Note: **: 2-sided *p*-value < 0.01; *: 2-sided *p*-value < 0.05. M/F = 0: only female; 0 < F/M < 1: fewer males than females; F/M > 1: more females than males.

## Data Availability

Data will be made available on request.

## Supplementary Materials

The following are available online at https://www.mdpi.com/article/10.3390/healthcare12171775/s1, Table S1: Preferred Reporting Items for Systematic Reviews and Meta-Analyses (PRISMA) statement.
